# Association Between p.R4810K Variant and Long-Term Clinical Outcome in Patients With Moyamoya Disease

**DOI:** 10.3389/fneur.2019.00662

**Published:** 2019-06-25

**Authors:** Peicong Ge, Xun Ye, Xingju Liu, Xiaofeng Deng, Rong Wang, Yan Zhang, Dong Zhang, Qian Zhang, Jizong Zhao

**Affiliations:** ^1^Department of Neurosurgery, Beijing Tiantan Hospital, Capital Medical University, Beijing, China; ^2^China National Clinical Research Center for Neurological Diseases, Beijing, China; ^3^Center of Stroke, Beijing Institute for Brain Disorders, Beijing, China; ^4^Beijing Key Laboratory of Translational Medicine for Cerebrovascular Disease, Beijing, China; ^5^Beijing Translational Engineering Center for 3D Printer in Clinical Neuroscience, Beijing, China; ^6^Savaid Medical School, University of Chinese Academy of Sciences, Beijing, China

**Keywords:** moyamoya disease, p.R4810K variant, clinical features, clinical outcome, stroke

## Abstract

**Objective:** To estimate the association between p. R4810K variant and clinical outcomes of patients with moyamoya disease (MMD).

**Methods:** The p.R4810K genetic variant was genotyped among 498 Chinese patients with MMD conducted from June 1, 2012, to June 31, 2017. Data was obtained by retrospective chart review, follow-up information and outcome were obtained through clinical visits and telephone.

**Results:** Among 498 patients, 361 (72.5%) were wild-type patients (G/G), 133 (26.7%) heterozygous patients (G/A), and 4 (0.8%) homozygotes (A/A). Compared with GG group, the patients in the G/A+A/A group were younger at diagnosis and had more familial cases, more transient ischemic attack cases, more posterior cerebral artery involved hemispheres, less unilateral lesions. After the median 53 months follow-up, strokes occurred in 9 patients in the G/A+A/A group and in 52 in the G/G group. Multivariate Cox regression analysis showed that the history of hypertension (HR, 2.294; 95% CI, 1.251–4.206; *p* = 0.007), the presence of TIA (HR, 0.319; 95% CI, 0.120–0.846; *p* = 0.022), and the Suzuki stage (HR, 1.510; 95% CI, 1.129–2.018; *p* = 0.005) were associated with recurrent stroke. The p.R4810K (HR, 0.601; 95% CI, 0.292–1.239; *p* = 0.168) was not associated with recurrent stroke. Multivariate logistic regression analysis showed that recurrent stroke (OR, 5.997; 95% CI, 2.583–13.924; *p* = 0.000) was the only factor associated with unfavorable neurological status. And the p.R4810K (OR, 0.885; 95% CI, 0.482–1.627; *p* = 0.695) was not associated with neurological status.

**Conclusions:** Compared to the patients in G/G group, patients in G/A+A/A group exhibited different clinical features, and had a lower rate of recurrent stroke and better clinical outcome after early medical and surgical interventions. Multivariate COX and logistic regression analysis showed that p.R4810Kvariant was not related to either recurrent stroke or neurological status. The p.R4810Kvariant may not be associated with long-term clinical outcome in Chinese patients with MMD.

## Introduction

Moyamoya disease (MMD) is a chronic cerebrovascular disorder (1). It can be characterized by the presence of the progressive occlusion which occurs at the terminal portions of the bilateral or unilateral carotid arteries and their main branches during the development of a basal collateral network (moyamoya vessels) (2). There are several clinical manifestations of MMD, such as infarction, hemorrhage, transient ischemia attack (TIA), headache, and seizures (3). Depending on the manifestation, there are mainly two phenotypes of MMD: the ischemic type and the hemorrhagic type (4).

Although MMD is a rare cerebrovascular disorder, it is the main cause of stroke in children and adolescents in the East Asian countries (3). Based on the surveys performed in recent years, the prevalence of MMD was approximately 6.03/100,000 in Japan, 16.1/100,000 in Korea and 3.92/100,000 in China (5–7). The cause of MMD remains unknown, but the RNF213 in the 17q25 has recently been identified as a susceptible gene, which may be a pathogenic gene mutation that leads to the development and progression of MMD in East Asian populations (8, 9). The c.14429G>A (p.R4810K) variant in RNF213 was identified as variants with a strong susceptibility in Asian patients with MMD (10). Understanding the relationship between p.R4810K variant and long-term clinical outcome is critically important to optimize treatment for patients with MMD. In this study, we investigated the association of long-term clinical outcomes and p.R4810K variant in MMD patients.

## Materials and Methods

### Patient Data

The study involved 508 MMD inpatients at Beijing Tiantan Hospital, Capital Medical University, from June 1, 2012, to June 31, 2017. MMD was diagnosed according to published guidelines for MMD (11). Patients with a history of cranial irradiation and meningitis, Down syndrome, brain tumors, and neurofibromatosis type 1 were excluded (12). Six patients with moyamoya syndrome (2 patients with a history of cranial irradiation for the germ cell tumor, 2 patients with a history of hypophysoma, 1 patient with down syndrome, and 1 patient neurofibromatiosis type 1) and four patients with incomplete imaging data were excluded. A total of 498 patients enrolled in this study. Blood samples were collected at admission. Sequencing methods for p.R4810K variant were reported in our previous study (13). The primers were designed as follows: RNF213-4810F (rs112735431):

5′-GCCCTCCATTTCTAGCACAC-3′; and RNF213-4810R: 5′-AGCTGTGGCGAAAGCTTCTA-3′. Information on patient sex, age at diagnosis, family history of MMD (at least one first or second degree relative with MMD), history of risk factors, including hypertension (a self-reported history of hypertension or use of any antihypertensive medication, systolic blood pressure ≥140 mmHg, or diastolic blood pressure ≥90 mmHg), smoking (a self-reported history), diabetes (a self-reported history of diabetes, or use of any hypoglycemic drugs, any fasting blood glucose level ≥7.0 mmol/L), alcohol use (self-reported information), aneurysm (diagnosed by DSA), hyperlipidemia (a self-reported history of hyperlipidemia, or use of any lipid-lowering medicine, low-density lipoprotein cholesterol ≥3.37 mmol/L, high-density lipoprotein cholesterol <1.04 mmol/L, triglycerides ≥1.7 mmol/L, or total cholesterol ≥5.17 mmol/L), thyroid disease (a self-reported history, or use of any antithyroid drugs), clinical manifestations including infarction, hemorrhage, transient ischemia attack (TIA), frequent TIAs (≥2 times per month), headache and seizures, and radiographic presentations at diagnosis, neurological status, and treatment was obtained by retrospective medical charts. Radiographic presentations including combined aneurysm, Suzuki stage (Table 1), posterior cerebral artery (PCA) involvement reviewed blindly by two neurosurgeons, any disagreement on the radiologic presentations was reevaluated by a third reader. This study has been approved by Beijing Tiantan Hospital research ethics committee. All participants or representatives provided written informed consent before being entered into the study.

**Table 1:** Suzuki stage.

**Suzuki stage**	**Angiographic features**
I	Narrowing of carotid fork, isolated narrowing of supraclinoid carotid
II	Initiation of the moyamoya, progressive narrowing of carotid, dilatation of native cerebral arteries, early formation of moyamoya vessels in basal carotid circulation
III	Intensification of the moyamoya vessels, In basal regions, exuberant moyamoya vessel formation, severe carotid stenosis with decreased flow in middle and anterior cerebral arteries
IV	Minimization of the moyamoya vessels, severe carotid stenosis with impaired filling of middle, anterior, and posterior cerebral arteries
V	Reduction of the moyamoya vessels, complete cessation of flow in ipsilateral middle, anterior, and posterior cerebral arteries
VI	Disappearance of moyamoya vessels, the internal carotid artery disappears completely, filling of cerebral vasculature by external carotid supply via leptomeningeal anastomoses

### Treatment and Follow-Up

The indication of surgical bypass for MMD at our institution was previously noted (14). Three types of surgical procedure was performed: including indirect bypass (IB), direct-bypass (DB), combined bypass (CB). DB and CB were the favored for most patients in our center, whether in children or adult patients, and IB was performed only when the donor or recipient artery was too small or fragile to perform artery anastomosis. Clinical outcome was conducted through clinical visits or telephone interview 3–6 months after discharge and annually thereafter. The medical charts were completed by the neurosurgeons who were blinded to the genotype of the patients. Follow-up events included TIA, ischemic stroke, intracranial hemorrhage, and death. New neurological deficit persisting for more than 24 h was defined as stroke. Infarction was defined by any new infarction on follow up brain imaging regardless of the presence of new neurological deficit. Medical management was optimized to treat vascular risk factors. Patients were maintained under antiplatelet therapy coverage (aspirin 100 mg/day). The modified Rankin Scale (mRS) score was collected at admission and in the follow up by telephone interview or personal clinical visits. Any improving of the patients' mRS score at admission after treatment was defined as “improvement in neurological status.”

### Statistical Analysis

Statistical analysis was conducted by using SPSS (Windows version 19.0, IBM). Continuous variables met the normal distribution were compared with the *t*-test, non-normally distributed continuous variables were compared with the rank-sum test, and categorical variables were compared with chi square test. The neurological status was dichotomized into favorable (mRS score <2) and unfavorable (mRS score ≥2, including death). The univariate and multivariable logistic regression analysis was used for evaluating the impact of clinical variables on the neurological status. The univariate and multivariate Cox regression was used to determine the significance of several variables for predicting the hazard ratio of recurrent stroke. Clinical variables achieving *P* < 0.05 in univariate analysis were included in multivariate analysis. All tests were 2-sided, and a *P*-value of 0.05 was defined to indicate statistical significance.

## Results

### Comparison of Clinical Characteristics With the RNF213 p.R4810K Genotype

We analyzed 498 patients (288 females and 210 males, Table 2). Of the 498 sequenced patients, 361 (72.5%) were wild-type patients (G/G), 133 (26.7%) heterozygous patients (G/A), and 4 (0.8%) homozygotes (A/A). The median age at diagnosis was significantly lower in the G/A+A/A group than in the G/G group (*p* = 0.000). Compared with the patients in the G/G group, patients in G/A+A/A group were included more familial cases (13.9 vs. 4.4%, *p* = 0.003), less cases had a history of hypertension (13.9 vs. 24.7%, *p* = 0.009), less cases suffered intracranial hemorrhage (18.2 vs. 29.1%, *p* = 0.014), more cases suffered TIA (32.8 vs. 22.2%, *p* = 0.014), less unilateral lesions (2.2 vs. 5.8%, *p* = 0.017) and more PCA involved hemispheres (31.4 vs. 16.6%, *p* = 0.000). There was no significant difference in the female/male ratio (81/55 vs. 207/154, *p* = 0.716), combined aneurysm (2.2 vs. 3.6%, *p* = 0.634), mRS score > 2 at admission (64.9 vs. 63.2%, *p* = 0.716), or conservative/revascularization ratio (3/134 vs. 17/344, *p* = 0.201) between two groups.

**Table 2:** Patient characteristics at admission.

**Characteristics**	**Total**	**G/A+A/A**	**G/G**	**χ2 or *Z***	***p*-Value**
No. of patients	498	137	361		
Female/male ratio	288/210	81/56	207/154	0.130	0.719
Age, median (IQR), y	33 (15–43)	25 (10–38)	35 (21–44)	−4.775	0.000
Age					
≤ 18 years (no. [%])	139 (27.9)	56 (40.9)	83 (23.0)	15.787	0.000
Family history (no. [%])	35 (7.0)	19 (13.9)	16 (4.4)	13.534	0.000
History of risk factors (no. [%])					
Hypertension	108 (21.6)	19 (13.9)	89 (24.7)	6.802	0.009
Smoking	30 (6.0)	4 (2.9)	26 (7.2)	2.505	0.113
Diabetes	27 (5.4)	3 (2.2)	24 (6.6)	3.029	0.082
Alcohol use	20 (4.0)	4 (2.9)	16 (4.4)	0.262	0.609
Hyperlipidemia	18 (3.6)	4 (2.9)	14 (3.9)	0.059	0.808
Aneurysm	16 (3.2)	3 (2.2)	13 (3.6)	0.263	0.608
Thyroid disease	10 (2.0)	2 (1.5)	8 (2.2)	0.032	0.858
Clinical manifestations (no. [%])					
Infarction	166 (33.3)	49 (35.8)	117 (32.4)	0.503	0.478
Hemorrhage	130 (26.1)	25 (18.2)	105 (29.1)	6.047	0.014
TIA	125 (25.1)	45 (32.8)	80 (22.2)	6.032	0.014
Frequent TIAs	44 (8.8)	10 (7.3)	34 (9.4)	0.554	0.457
Headache	19 (3.8)	4 (2.9)	15 (4.2)	0.145	0.703
Seizures	14 (2.8)	4 (2.9)	10 (2.8)	0.000	1.000
Bilateral lesions (no. [%])	450 (90.4)	131 (95.6)	319 (88.4)	6.001	0.014
Suzuki stage^*^					
0 (no. [%])	47 (4.8)	6 (2.2)	41 (5.8)	5.694	0.017
1–2 (no. [%])	146 (14.9)	37 (13.5)	109 (15.5)	0.608	0.435
3–4 (no. [%])	658 (67.3)	195 (71.2)	463 (65.8)	2.613	0.106
5–6 (no. [%])	127 (13.0)	36 (13.1)	91 (12.9)	0.008	0.929
PCA involved hemispheres^*^	201 (20.5)	86(31.4)	117 (16.6)	26.151	0.000
mRS score <2 at admission	317 (63.7)	88 (64.2)	228 (63.2)	0.050	0.824
Conservative/surgical ratio	20/478	3/134	17/344	1.635	0.201

*A/A, homozygous patients; G/A, heterozygous patients; G/G, wild-type patients; IQR, interquartile range; mRS, modified Rankin Scale; PCA, posterior cerebral artery; TIA, transient ischemia attack*.

* *489 MMD patients (978 hemispheres) received cerebral angiograph*.

### Analysis for Predictive Factors for Recurrent Stroke

In the G/A+A/A group, 134 patients (97.8%) received revascularization surgery, including 144 IB, 52 DB and 18 CB. In the G/G group, 361 patients (95.2%) underwent revascularization surgery, including 267 CB procedures, 185 DB procedures and 43 IB procedures. In the follow-up, a total of 7 patients (1.4%)−1 in the G/A+A/A group and 6 in the G/G group—were lost to follow-up, 491 patients were analyzed (Table 3). During the median 53 months follow-up after discharge, strokes occurred in 9 patients (6.6%, including 3 perioperative strokes) in the G/A+A/A group and in 52 (14.6%, including 21 perioperative strokes) in the G/G group. Univariate Cox regression analysis showed that the p.R4810K mutation (HR, 0.454; 95% CI, 0.224–0.921; *p* = 0.029) and the presence of TIA (HR, 0.300; 95% CI, 0.129–0.698; *p* = 0.005) were associated with lower risk of recurrent stroke (Table 4). On the other hand, the history of hypertension (HR, 3.428; 95% CI, 2.070–5.677; *p* = 0.000), the history of hyperlipidemia (HR, 2.984; 95% CI, 1.080–8.242; *p* = 0.035), the presence of infarction (HR, 1.888; 95% CI, 1.142–3.120; *p* = 0.013), and the advanced Suzuki stage (HR, 1.565; 95% CI, 1.176–2.083; *p* = 0.002) were associated with higher risk of recurrent stroke. Multivariate Cox regression analysis showed that the history of hypertension (HR, 2.294; 95% CI, 1.251–4.206; *p* = 0.007), the presence of TIA (HR, 0.319; 95% CI, 0.120–0.846; *p* = 0.022), and the Suzuki stage (HR, 1.510; 95% CI, 1.129–2.018; *p* = 0.005) were associated with recurrent stroke. And the p.R4810K (HR, 0.601; 95% CI, 0.292–1.239; *p* = 0.168) was not associated with recurrent stroke.

**Table 3:** Association of long-term outcomes with the c.14429G>A (p.R4810K) genotype of RNF213 in 491 patients with MMD.

**Outcome**	**G/A+A/A (*n* = 136)**	**G/G (*n* = 355)**	**χ2 or *Z***	***p*-Value**
Baseline data				
Sex ratio (female/male)	81/55	205/150	0.133	0.716
Age, median (IQR), y	25 (10–38)	35 (21–44)	−4.647	0.000
Family history (no. [%])	18 (13.2)	16 (4.5)	11.623	0.001
History of risk factors (no. [%])				
Hypertension	19 (14.0)	88 (24.8)	6.752	0.009
Diabetes	3 (2.2)	24 (6.8)	3.098	0.078
Hyperlipidemia	4 (2.9)	13 (3.7)	0.013	0.908
Smoking	4 (2.9)	25 (7.0)	2.284	0.131
Alcohol use	4 (2.9)	16 (4.5)	0.281	0.596
Thyroid disease	2 (1.5)	7 (2.0)	0.000	1.000
Follow-up, median (IQR), m	51 (31–62)	53 (35–62)	−1.055	0.291
Follow-up events (no. [%])				
TIAs	9 (6.6)	7 (2.0)	6.732	0.009
Ischemic stroke	3 (2.2)	12 (3.4)	0.147	0.701
Hemorrhagic stroke	3 (2.2)	19 (5.4)	1.599	0.206
Neurological status (no. [%])				
Improvement in mRS score	98 (72.1)	218 (61.4)	4.862	0.023
mRS Score 0–1	124 (91.2)	289 (81.4)	7.021	0.008
mRS Score 2–5	10 (7.4)	57 (16.1)	6.321	0.012
Vascular death	2 (1.5)	9 (2.5)	0.139	0.709

*A/A, homozygous patients; G/A, heterozygous patients; G/G, wild-type patients; IQR, interquartile range; mRS, modified Rankin Scale; TIA, transient ischemia attack*.

**Table 4:** The COX regression analysis of predictors for the recurrent stroke.

**Characteristics OR (95%CI)**	**Univariate**		**Multivariate**	
	***p*-Value**	**HR (95% CI)**	***p*-Value**	**HR (95% CI)**
p.R4810K	**0.029**	0.454 (0.224–0.921)	0.168	0.601 (0.292–1.239)
Female sex	0.550	0.858 (0.518–1.419)		
Age	**0.000**	1.019 (1.002–1.036)	0.144	1.017 (0.994–1.039)
Family history	0.493	0.666 (0.209–2.218)		
History of risk factors				
Hypertension	**0.000**	3.428 (2.070–5.677)	**0.007**	2.294 (1.251–4.206)
Smoking	0.782	1.154 (0.308–2.700)		
Diabetes	0.074	2.160 (0.929–5.020)		
Alcohol use	0.264	1.783(0.647–4.917)		
Hyperlipidemia	**0.035**	2.984 (1.080–8.242)	0.903	0.928 (0.279–3.084)
Aneurysm	0.255	1.961 (0.615–6.257)		
Thyroid disease	0.321	2.042 (0.499–8.361)		
Clinical manifestation				
Infarction	**0.013**	1.888 (1.142–3.120)	0.624	1.147 (0.662–1.986)
Hemorrhage	0.067	1.631 (0.967–2.751)		
TIA	**0.005**	0.300 (0.129–0.698)	**0.022**	0.319 (0.120–0.846)
Frequent TIAs	0.382	0.596 (0.187–1.903)		
Headache	0.264	0.046 (0.000–10.108)		
Seizures	0.598	0.588 (0.081–4.242)		
Suzuki stage	**0.002**	1.565 (1.176–2.083)	**0.005**	1.510 (1.129–2.018)
PCA involved	0.535	0.827 (0.454–1.506)		
Surgery	0.310	21.330 (0.058–7818.146)		

*Boldface indicates statistical significance (p < 0.05). CI, confidence intervals; HR, hazard ratio; PCA, posterior cerebral artery; TIA, transient ischemia attack*.

### Analysis for Predictive Factors for the Neurological Status

Over the median 53 months long-term follow-up after discharge, favorable neurological status (mRS <2) was observed in 413 patients (84.1%) and unfavorable neurological status (mRS≥2) was observed in 78 patients (15.9%). An improvement in neurological status was found in 98 (72.1%) of 136 in the G/A+A/A group and 218 (61.4%) of 355 in the G/G group (*p* = 0.027). Vascular death (including death from hemorrhagic stroke) occurred in 2 patients (1.5%) in the G/A+A/A group and 9 (2.5%) in the G/G group. The univariable logistic regression showed that p.R4810K (OR, 0.832; 95% CI, 0.469–1.476; *p* = 0.530) was not associated with long-term neurological status (Table 5). The increasing age (OR, 1.019; 95% CI, 1.002–1.036; *p* = 0.031) was associated with a higher risk of unfavorable neurological status. And recurrent stroke (OR, 2.580; 95% CI, 1.379–4.827; *p* = 0.003) was also associated with unfavorable neurological status. Furthermore, multivariate logistic regression analysis showed that recurrent stroke (OR, 5.997; 95% CI, 2.583–13.924; *p* = 0.000) was the only factor associated with neurological status. And the p.R4810K (OR, 0.885; 95% CI, 0.482–1.627; *p* = 0.695) was not associated with neurological status.

**Table 5:** Logistic regression analysis of predictors for the neurological status.

**Characteristics OR (95%CI)**	**Univariate**		**Multivariate**	
	***p*-Value**	**OR (95% CI)**	***p*-Value**	**OR (95% CI)**
p.R4810K	0.530	0.832 (0.469–1.476)	0.695	0.885 (0.482–1.627)
Female sex	0.250	1.354 (0.808–2.270)		
Age	**0.031**	1.019 (1.002–1.036)	0.071	1.016 (0.999–1.035)
Family history	0.580	1.297 (0.516–3.260)		
History of risk factors				
Hypertension	0.120	1.559 (0.891–2.728)		
Smoking	0.867	0.911 (0.308–2.700)		
Diabetes	0.574	0.704 (0.206–2.400)		
Alcohol use	0.986	1.011(0.289–3.540)		
Hyperlipidemia	0.423	0.433 (0.056–3.359)		
Aneurysm	0.255	1.961 (0.615–6.257)		
Thyroid disease	0.626	2.943 (0.719–12.040)		
Clinical manifestation				
Infarction	0.868	1.045 (0.619–1.766)		
Hemorrhage	0.086	1.593 (0.936–2.712)		
TIA	0.198	0.664 (0.357–1.238)		
Frequent TIAs	0.912	0.950 (0.385–2.343)		
Headache	0.590	0.664 (0.250–2.939)		
Seizures	0.423	0.433 (0.056–3.359)		
Suzuki stage	0.976	1.004 (0.757–1.332)		
PCA involved	0.119	0.610 (0.327–1.136)		
Surgery^*^	0.376	0.598 (1.191–1.869)	0.426	0.620 (0.191–2.011)
Recurrent Stroke	**0.003**	2.580(1.379–4.827)	**0.000**	5.997 (2.583–13.924)

* *Adjusted for surgery. Boldface indicates statistical significance (p < 0.05)*.

*CI, confidence intervals; OR, odds ratio; PCA, posterior cerebral artery; TIA, transient ischemia attack*.

## Discussion

The RNF213 in the 17q25 has recently been identified as a susceptible gene possible causative gene mutation leading to the development as well as progression of MMD in East Asian populations (15, 16). There is strong association between p.R4810K variant in RNF213 and MMD (10, 17–20). It was reported p.R4810K variant was identified in 90% of Japanese patients, 79% of Korean patients, and 23% of Chinese patients (17–19). In our study, p.R4810K variant was identified in 137 patients (27.5%), which was much lower than the Japanese and Korean patients.

Phenotype-genotype (p.R4810K) correlation in MMD has been well-documented. In Japanese patients, compared with heterozygous or wild-type patients, homozygous patients were earlier age at onset. Infarction, PCA involvement at the initial onset and the frequency of homozygotes was significantly higher than that of heterozygotes and wild types (18). In Korean patients, homozygous patients were also earlier age at onset than other types and all homozygous patients manifested MMD at age <5 years. Infarctions at initial presentation was also more frequent in the homozygous patients (17). In our study, only four patients (0.8%) were homozygous, which was much lower than Japanese and Korean patients (17, 18). What‘s more, compared with G/G group, more cases in G/A+A/A group suffered TIA (32.8 vs. 22.2%, *p* = 0.014), which was different from the previous studies (17, 18).

The angiography of patients with and without the RNF213 mutation was different (17, 18, 21). Miyatake et al. reported that bilateral vasculopathy was shown significant difference between wild-type and other genotypes (18). In our study, we found that unilateral MMD patients were more in the G/G group. In addition, our previous study also noted that p.R4810K variant was identified only 11.8% in unilateral MMD (22). Recently Kim et al reported that patients with RNF213 gene variants had more cases PCA involved and fewer leptomeningeal collateral circulation compensation from the PCA to anterior circulation (21). In this study, we found that more cases PCA involved in the G/G+AA group than the wild-type group, which was similar to the previous reports (17, 18, 21).

Clinical outcome-genotype (p.R4810K) correlation remains unknown. Although the homozygous variant was associated with early-onset MMD and infarction at diagnosis, known as poor prognostic factors for MMD, but it is lack of long-term follow-up results (17, 18). Comparing with the G/G group, patients in G/A+AA group, had a lower stroke recurrence rate and more improvement in mRS score. Kim et al. found that RNF213-positive cases had better post-operative collateral formation than RNF213-negative cases, which indicated that RNF213-positive cases may have a lower stroke recurrence rate and better clinical outcomes after the revascularization (21). The increased expression of angiogenic factors was observed both serum and cerebrospinal fluid of MMD patients (23, 24). On the contrary, cellular experiment and animal study showed that angiogenesis was impaired in the patients with p.R4810K mutation (23, 25). And recent study also showed that p.R4810K may not associated with the long-term clinical manifestations or poor prognosis in Japanese patients with MMD (26). In this study, we focused on the long-term clinical outcome-genotype correlation. In our study, although univariate Cox regression analysis showed that the p.R4810K mutation had lower recurrent stroke, multivariate Cox regression analysis showed that p.R4810K was not associated with recurrent stroke. What‘s more, both univariate and multivariate logistic regression analysis also showed that p.R4810K variant was not associated with long-term neurological status. Multivariate logistic regression analysis showed that recurrent stroke was the only factor associated with unfavorable neurological status. The p.R4810K variant was not associated with either recurrent stroke or neurological status.

### Limitation

Here are the limitations of our study. First, this study was a non-randomized retrospective study, despite a relatively large sample size for estimating the clinical outcome-genotype correlation in MMD, the power for detecting some associations may have been limited. Second, the study may have a selection bias because all the patients of our study were chosen from one neurosurgery center. Third, most of patients included in our study received surgical treatment, limited number of patients received conservative therapy, this study may not explain the prognosis of patients who underwent conservative therapy. Fourth, only p.R4810K variant rather than the RNF213 exons or whole gene were examined. Therefore, comprehensive genetic analysis of RNF213 is necessary to determine whether other RNF213 variants influence the long-term outcome of MMD.

## Conclusion

Compared to the patients in G/G group, the patients in G/A+A/A group were younger at diagnosis and had more familial cases, more transient ischemic attack cases, more posterior cerebral artery involved hemispheres, less unilateral lesions. Multivariate COX and logistic regression analysis showed that p.R4810Kvariant was not related to either recurrent stroke or neurological status. The p.R4810Kvariant may not be associated with long-term clinical outcome in Chinese patients with MMD.

## Ethics Statement

This study was carried out in accordance with the recommendations of Guideline set by Research Committee on Spontaneous Occlusion of the Circle of Willis, the ethics committee of Beijing. Tiantan Hospital with written informed consent from all subjects. All subjects gave written informed consent in accordance with the Declaration of Helsinki. The protocol was approved by the ethics committee of Beijing Tiantan Hospital, Capital Medical University.

## Author Contributions

PG and QZ: conception and design. PG, XY, XL, and XD: acquisition of data. PG and QZ: analysis and interpretation of data. PG: drafting the article. RW, YZ, and DZ: technical supports and surgery. All authors critically revising the article and reviewed submitted version of manuscript. JZ: approved the final version of the manuscript on behalf of all authors. JZ and QZ: study supervision.

### Conflict of Interest Statement

The authors declare that the research was conducted in the absence of any commercial or financial relationships that could be construed as a potential conflict of interest.
